# Adaptive bi-level programming for optimal gene knockouts for targeted overproduction under phenotypic constraints

**DOI:** 10.1186/1471-2105-14-S2-S17

**Published:** 2013-01-21

**Authors:** Shaogang Ren, Bo Zeng, Xiaoning Qian

**Affiliations:** 1 Department of Computer Science and Engineering, University of South Florida, Tampa, FL 33620, USA; 2 Department of Industrial and Management Systems Engineering, University of South Florida, Tampa, FL 33620, USA

## Abstract

**Background:**

Optimization procedures to identify gene knockouts for targeted biochemical overproduction have been widely in use in modern metabolic engineering. Flux balance analysis (FBA) framework has provided conceptual simplifications for genome-scale dynamic analysis at steady states. Based on FBA, many current optimization methods for targeted bio-productions have been developed under the maximum cell growth assumption. The optimization problem to derive gene knockout strategies recently has been formulated as a bi-level programming problem in OptKnock for maximum targeted bio-productions with maximum growth rates. However, it has been shown that knockout mutants in fact reach the steady states with the minimization of metabolic adjustment (MOMA) from the corresponding wild-type strains instead of having maximal growth rates after genetic or metabolic intervention. In this work, we propose a new bi-level computational framework--MOMAKnock--which can derive robust knockout strategies under the MOMA flux distribution approximation.

**Methods:**

In this new bi-level optimization framework, we aim to maximize the production of targeted chemicals by identifying candidate knockout genes or reactions under phenotypic constraints approximated by the MOMA assumption. Hence, the targeted chemical production is the primary objective of MOMAKnock while the MOMA assumption is formulated as the inner problem of constraining the knockout metabolic flux to be as close as possible to the steady-state phenotypes of wide-type strains. As this new inner problem becomes a quadratic programming problem, a novel adaptive piecewise linearization algorithm is developed in this paper to obtain the exact optimal solution to this new bi-level integer quadratic programming problem for MOMAKnock.

**Results:**

Our new MOMAKnock model and the adaptive piecewise linearization solution algorithm are tested with a small *E. coli *core metabolic network and a large-scale iAF1260 *E. coli *metabolic network. The derived knockout strategies are compared with those from OptKnock. Our preliminary experimental results show that MOMAKnock can provide improved targeted productions with more robust knockout strategies.

## Introduction

Metabolic engineering has become an important environment friendly process in modern biotechnology, providing new potential solutions to many global problems, including energy and environmental crisis [1-5]. Metabolic engineering improves wild-type strains, typically from microbial organisms, by searching for metabolically or genetically engineered strains for the optimal yields of bio-based productions including industrial and pharmaceutical chemicals, for example, bioethanol [3], bioplastics [6], and many synthesized amino acids like succinic acid [7] for alternative energy resources, cosmetics and pharmaceuticals with competitive cost compared to traditional chemistry-based technologies.

Classical metabolic engineering modifies individual metabolic genes or pathways, typically followed by costly and time-consuming screening processes to select desirable mutants based on their resulting phenotypes [8]. The recent unprecedented advent of high-throughput omics technologies has enabled more rational and effective metabolic engineering at systems level with a global understanding of biological systems, leading to a promising new discipline--"genome-scale synthetic biology" [9]. Integrated with computational modeling approaches, genome-scale metabolic network models [10], capturing coordinated interactions in cells, have made *in silico *whole-cell simulations possible to identify globally optimal targets for metabolic engineering with accurately predicted phenotypes [11-13].

*In silico *genome-scale manipulation of metabolism requires accurate metabolic flux dynamic analysis. Flux balance analysis (FBA) framework [14] has laid the foundation for many computational methods in metabolic engineering. In FBA, the constraints imposed by stoichiometry at metabolic flux steady states can be concisely captured with a mathematical linear model for balanced production and consumption fluxes. Based on this framework, several metabolic flux distribution approximation models have been proposed by the researchers. The first simplified model assumes that cells metabolize for maximum growth at steady states, which naturally leads to the biomass maximization model proposed in [15]. In [11], the authors point out that knockout metabolic fluxes undergo a minimization of metabolic adjustment (MOMA) process rather than directly heading to the maximizing biomass state without being exposed to long-term evolutionary pressure. The simulation results based on this model have shown better agreement with observations in experiments with knockout strains. Another model named regulatory on/off minimization (ROOM) [12] has been proposed to address the long-term post knockout metabolic flux distribution predication problem. The ROOM model is still based on the assumption that the underlying regulatory mechanisms in cells aim to minimize flux changes after genetic perturbations but constraining on the number of reactions with large flux changes.

Researchers have proposed different metabolic engineering methods based on these metabolic approximation models and typically the improved strains are sequentially modified based on FBA with multiple mutants. However, sequential metabolic engineering strategies do not have the guarantee of the optimality. In [13], the authors have introduced the OptKnock framework for suggesting gene deletion strategies for the optimal overproduction of specific chemical compounds based on the biomass maximization assumption. OptKnock is formulated as a bi-level programming problem. Its primal objective is to maximize the overproduction of targeted bio-productions at the first or outer level under the condition that cells are still live, which is modeled as the second or inner level optimization problem of maximizing the cell growth, approximated by the assumption of maximization of biomass yields. Although the biomass maximization assumption for wild-type strains is justifiable, the same argument may not be valid for engineered knockouts or other microbial strains that were not exposed to long-term evolutionary pressure [11]. Without enough constraints on the resulting flux distributions, OptKnock may generate impractical knockout strategies. As the MOMA assumption provides more strict phenotypic constraints to steady-state fluxes for engineered knockout strains with validated congruency with experimental observations, it may provide better constraints to knockout steady-state flux distributions to systematically search for more realistic knockout strategies in given metabolic network models.

In this paper, we propose a bi-level programming framework for the identification of optimal genetic manipulations under the MOMA assumption. With the new MOMA assumption to approximate the condition to maintain the cell liveness as the essential phenotypic constraints, the inner optimization problem becomes a quadratic programming (QP) problem rather than the linear programming (LP) problem in OptKnock. To address the raised computational complexity, we develop a novel adaptive solution algorithm to solve this new bi-level optimization problem. The new algorithm under the minimizing flux adjustment assumption is tested on metabolic networks and our preliminary experimental results show that our framework can generate more practical and robust knockout strategies compared to OptKnock.

## Methods

### Backgrounds: FBA and MOMA

Before introducing our new bi-level programming problem to identify optimal metabolic genes or reactions to delete for the maximization of targeted bio-productions, we first review the mathematical foundations of FBA [14] and MOMA [11]. FBA provides appropriate simplifications for metabolic flux analysis by assuming the balance of production and consumption fluxes at steady states of metabolic network models. Specifically, with the prior stoichiometry knowledge, FBA assumes that the weighted sum of network fluxes based on stoichiometric coefficients *S *is 0: $\sum_{j=1}^{M}S_{ij}v_{j}=0$, 1 ≤ *i *≤ *N*, in which we assume that the network model has *M *reactions and *N *metabolites in total; *S_ij _*is the stoichiometric coefficient of metabolite *i *in reaction *j*; and *v_j _*denotes the flux value of reaction *j*. For wild-type strains, as mentioned above, a common assumption is that their steady-state flux values follow an optimal distribution that maximizes the biomass production rate. The steady-state flux distribution is approximately solved as a LP problem to maximize the biomass production flux: ${\text{max}}_{v_{j,1\leq j\leq M}}$*v_biom _*subject to the FBA stoichiometry constraints, in which *v_biom _*is defined by summing up the metabolite precursors that contribute to the biomass production in FBA [11]. In OptKnock, the optimal gene knockout strategy is to remove genes or reactions by setting the corresponding *v_j _*to zero with the resulting knockout flux distribution maintaining biomass maximization assumption.

As stated in [11], engineered gene knockouts in laboratory usually cannot achieve the maximum growth states as they have not been exposed to the same evolutionary pressure as wild-type strains. Typically, mutant strains initially stay as close as possible to wild-type optimal steady states in terms of flux values. Computational simulations under the MOMA assumption constraining metabolic adjustment to be minimal have demonstrated better agreement with observed flux values in actual experiments [11]. Hence, flux distributions in mutated metabolic networks can be solved as a QP optimization problem to minimize the *L*_2 _distance between the knockout flux values to wild-type steady-state flux values:

$$\begin{array}{c} \underset{v}{\text{min}}\underset{j}{\sum }{\left(v_{j}-w_{j}\right)}^{2}\\ s.t.\underset{j}{\sum }S_{ij}v_{j}=0\;,\forall i\\ v_{glc}=v_{glc\text{\_}uptake}\\ v_{biom}\geq v_{biom}^{target}\\ v_{j}^{min}\leq v_{j}\leq v_{j}^{max},\forall j \end{array}$$

where *v_j _*represents the flux value of reaction *j *in mutant strains and *w_j _*is the corresponding flux value in wild-type strains. The flux value for biomass production *v_biom _*is similarly defined as mentioned earlier. In addition, the glucose flux value *v_glc _*denotes the glucose consumption rate, which is often set to a fixed value *v_glc_uptake_*. Finally, $v_{j}^{min}$ and $v_{j}^{max}$ are the lower bound and upper bound for *v_j_*, which are determined by the availability of nutrients or the maximal fluxes that can be supported by enzymatic pathways [11].

### New bi-level programming framework

Following the modeling strategy in OptKnock [13], we aim to derive optimal gene knockout strategies, which consequently remove corresponding reactions for desired biomedical overproduction while maintaining obligatory cellular conditions, for example, cell mortality. However, as it has been shown that the assumption of biomass maximization for steady-state cellular conditions may not correctly predict metabolic flux distributions for knockouts [11,13], we replace the internal cellular objective of maximizing biomass yield in OptKnock [13] by the MOMA assumption [11], which has led to better predictions of steady-state flux allocations for genetically engineered strains. With this critical change from OptKnock, we formulate a novel bi-level programming model for gene knockouts in which the inner optimization problem is a QP problem.

Mathematically, we introduce binary variables *y_j _*∈ {0, 1}, 1 ≤ *j *≤ *M*, denoting gene or reaction knockout strategies in which reaction *j *either is knocked out (*y_j _*= 0) or remains active (*y_j _*= 1). The identification of optimal knockout strategies *y_j _*under MOMA requires to solve the following bi-level programming problem:

$$\begin{array}{c} \underset{y}{\text{max}}v_{chemical}\\ s.t.\left\{\begin{array}{c} \underset{v}{\text{min}}\underset{j:j\neq chemical}{\sum }{\left(v_{j}-w_{j}\right)}^{2}\\ s.t.\underset{j}{\sum }S_{ij}v_{j}=0,\;\forall i\\ v_{glc}=v_{glc\text{\_}uptake}\\ v_{biom}\geq v_{biom}^{target}\\ v_{j}^{min}\cdot y_{j}\leq v_{j}\leq v_{j}^{max}\cdot y_{j},\forall j \end{array}\right)\\ \underset{j}{\sum }\left(1-y_{j}\right)\leq K\\ y_{j}=\left\{0,1\right\}, \end{array}$$

in which *K *is the allowed maximum number of knockouts and *v_chemical _*corresponds to the reaction that produces the desired biochemical production target. Note that we do not count in the flux change for the target reaction in the inner problem as it would contradicts to our primal optimization for maximal biochemical overproduction.

### Adaptive linearization strategy for an exact optimal solution

We emphasize that the nested inner optimization problem is a QP problem with respect to flux allocation *v_j _*in knockout strains. As this nested inner problem is convex, we can still get its dual problem and the strong duality condition still holds for the inner primal and dual problems. Following the similar direction of [13], we can develop a single-level equivalent formulation by enforcing the objective value of the inner primal problem equal to that of its dual problem. However, the resulting formulation will be a mixed integer quadratically constrained programming problem, which poses a huge computational challenge when solving real problems. Because of this major change due to the introduction of the inner QP problem under the MOMA assumption, the transformation in OptKnock to a typical single-level mixed integer linear programming (MILP) problem based on the linear programming (LP) duality theory is not directly applicable any more.

**Figure 1:**
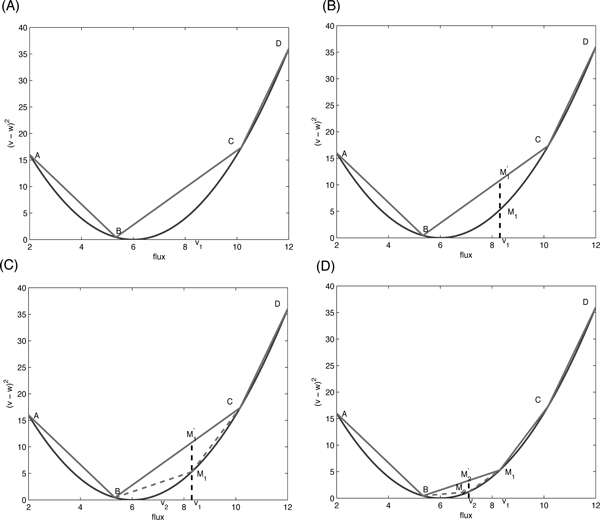
**Schematic illustration of adaptive linearization solution strategy to the new bi-level programming problem under the MOMA assumption: (A) Piecewise linearization; (B-D) Adaptive solution strategy**.

To derive efficient solution algorithms for our new bi-level programming gene knockout problem, we adopt a novel adaptive linearization solution strategy to tackle the computational complexity introduced by the inner QP problem. Specifically, we propose to adaptively represent the quadratic terms in the objective function of the inner problem using a set of linear functions as illustrated in Figure 1(A), which yields a LP approximation for the nested inner problem. With a given piecewise linearization of the inner problem, we can convert our *new bi-level model *into a single-level problem based on the LP strong duality. For the linearized problem, we can obtain the optimal solution similarly as in [13] by solving the transformed single-level MILP problem. In order to obtain the exact optimal solution to the original bi-level problem with the inner QP problem, we adaptively create necessary pieces on the fly to approximate the quadratic objective function until the solution converges.

The basic idea of adaptive piecewise linearization is illustrated in Figure 1(B-D). We denote the initial starting solution by *v*_1_, which can be represented by a convex combination of endpoints of piecewise segments for a given piecewise linearization. The corresponding quadratic objective function value at *v*_1 _is denoted by *M*_1_, which can be approximated linearly by $M_{1}^{\prime }$as the convex combination of the corresponding objective function values at segment endpoints A, B, C and D. We iterate the procedures to solve the linearized single-level MILP problem and to adaptively add piecewise linear segments to better approximate the inner quadratic objective function as illustrated in Figure 1(B-D) until the optimal solution of the MILP problem achieves the desired precision with respect to the approximation of the inner QP objective function. This adaptive linearization strategy has the guarantee that the final solution converges to the exact optimal solution. More importantly, it is much more efficient than directly solving mixed integer quadratic constrained problem without linearization and hence it allows us to solve for large-scale metabolic networks.

With this basic understanding of our new bi-level model and adaptive piecewise linearization solution strategy, we describe the detailed algorithm in the following sections.

### Piecewise linearized inner problem

The quadratic objective function of the inner problem, denoting the metabolic adjustment to wild-type steady-state flux allocations (*w_j_*) in MOMA, is the key obstacle to derive the efficient solution strategy. We propose to use piecewise linear functions to approximate this quadratic objective function. The basic idea of piecewise linearization is to assume that each reaction flux value *v_j _*can be discretized into a finite number of segments, each of which is precisely defined by its corresponding consecutive endpoints $\left(v_{j}^{t},v_{j}^{t+1}\right)$. Any arbitrary value *v_j _*can then be represented by a convex combination of these endpoints:

(1) $$v_{j}=\overset{T}{\underset{t=1}{\sum }}\beta_{j}^{t}v_{j}^{t},$$

in which $\beta_{j}^{t}$ are the piecewise variables determining the convex representation and there are *T *- 1 segments with *T *endpoints between the corresponding lower and upper bounds for flux value *v_j_*: $v_{j}^{min}$ and $v_{j}^{max}$. These piecewise variables $\beta_{j}^{t}$ satisfy the following constraints to guarantee the satisfaction of the flux constraints $v_{j}^{min}\leq v_{j}\leq v_{j}^{max}$:

(2) $$\overset{T}{\underset{t=1}{\sum }}\beta_{j}^{t}=1,\;\forall j;$$

(3) $$\beta_{j}^{t}\geq 0,\;\forall j,t.$$

Similarly, as can be seen from Figure 1, the individual contribution from flux *v_j _*to the original quadratic objective function of the inner problem can be approximated as

(4) $$\begin{array}{ccc} {\left(v_{j}-w_{j}\right)}^{2} & \approx \overset{T}{\underset{t=1}{\sum }}{\left(v_{j}^{t}-w_{j}\right)}^{2}\cdot \beta_{j}^{t} & \\ & =\overset{T}{\underset{t=1}{\sum }}\left(v_{j}^{t^{2}}-2w_{j}\cdot v_{j}^{t}\right)\cdot \beta_{j}^{t}+w_{j}^{2}. \end{array}$$

With this convex approximation strategy, the inner problem with MOMA is transformed to a linear programming problem with respect to the piecewise variables $\beta_{j}^{t}$:

$$\begin{array}{c} \underset{\beta }{\text{min}}\;\underset{j:j\neq chemical}{\sum }\underset{t}{\sum }\left(v_{j}^{t^{2}}-2w_{j}\cdot \;v_{j}^{t}\right)\cdot \beta_{j}^{t}\\ s.t.\;\underset{t}{\sum }\beta_{j}^{t}=1\;\;\forall j;\\ \;\;\underset{j}{\sum }\underset{t}{\sum }\;S_{ij}v_{j}^{t}\beta_{j}^{t}=0\;\;\forall i;\\ \;\;\underset{t}{\sum }v_{glc}^{t}\beta_{glc}^{t}={v_{glc}}_{\text{\_}uptake};\;\;\underset{t}{\sum }\overset{t}{\underset{biom}{v}}\overset{t}{\underset{biom}{\beta }}\geq v_{biom}^{target};\\ \;\;\underset{t}{\sum }-v_{j}^{t}\beta_{j}^{t}\geq -v_{j}^{max}\cdot y_{j}\;\forall_{j};\;\;\underset{t}{\sum }v_{j}^{t}\beta_{j}^{t}\geq v_{j}^{min}\cdot y_{j}\;\forall j;\\ \;\;\beta_{j}^{t}\geq 0\;\;\forall j,\;t. \end{array}$$

Here, both *w_j _*and $v_{j}^{t}$ are constants and we have removed the constant terms $w_{j}^{2}$ in the original objective function. This linear approximation of the original inner objective function based on the MOMA criterion now enables the solution strategy to the bi-level programming problem by taking advantage of the LP strong duality property [16], for which the objective function values for the primal and dual problems of the approximated inner LP problem must be equal to each other at optimality if both of them are bounded. With this duality condition, the bi-level programming problem can be solved as a single-level MILP problem by including the dual problem formulation and enforcing that the primal and dual problems share the same objective function value as in [13].

We first give the dual problem of the linearized inner problem:

$$\begin{array}{c} \underset{a,b,\mu_{glc},\mu_{biom},c,d}{\text{max}}\underset{j}{\sum }a_{j}+v_{glc_{-}uptake}\mu_{glc}+v_{biom}^{target}\mu_{biom}-\underset{j}{\sum }v_{j}^{min}c_{j}y_{j}+\underset{j}{\sum }v_{j}^{max}d_{j}y_{j}\\ s.t.\;a_{j}+\underset{i}{\sum }S_{ij}v_{j}^{t}b_{i}-v_{j}^{t}c_{j}+v_{j}^{t}d_{j}\leq v_{j}^{t^{2}}-2w_{j}v_{j}^{t}\;\;\forall j,t,j\neq glc,\;biom,\;chemical;\\ \;v_{glc}^{t}\mu_{glc}+a_{glc}+\underset{i}{\sum }S_{i,glc}v_{glc}^{t}b_{i}-v_{glc}^{t}c_{glc}+v_{glc}^{t}d_{glc}\leq {v_{glc}^{t}}^{2}-2w_{glc}v_{glc}^{t}\forall t;\\ \;v_{biom}^{t}\mu_{biom}+a_{biom}+\underset{i}{\sum }S_{i,biom}v_{biom}^{t}b_{i}-v_{biom}^{t}c_{biom}+v_{biom}^{t}d_{biom}\leq {v_{biom}^{t}}^{2}-2w_{biom}v_{biom}^{t}\forall t;\\ \;a_{chemical}+\underset{i}{\sum }S_{i,chemical}v_{chemical}^{t}b_{i}-v_{chemical}^{t}c_{chemical}+v_{chemical}^{t}d_{chemical}\leq 0\;\;\forall t;\\ \;\mu_{biomass}\geq 0,\;c_{j}\geq 0,\;d_{j}\geq 0\;\;\forall j, \end{array}$$

where *a_j _*is the corresponding dual variable associated with the constraints on new piecewise variables *β*; *b_i _*is the dual variable for stoichiometric constraints, *c_j _*and *d_j _*are the dual variables for upper bound constraints and lower bound constraints for flux values respectively, and *µ_glc _*and *µ_biom _*are the dual variables corresponding to the constraints for glucose and biomass flux values. The knockout variable *y_j _*is still in the inner dual problem coupling two cellular objectives in the original outer and inner problems. The products of single binary variable and continuous variable in the fourth and the fifth terms can be linearized using the big-M method. Together with LP duality constraint, we have the final single-level MILP problem as

$$\begin{array}{c} \underset{y}{\text{max}}\underset{t}{\sum }v_{chemical}^{t}\beta_{chemical}^{t}\\ s.t\underset{j:j\neq chemical}{\sum }\underset{t}{\sum }\left({v_{j}^{t}}^{2}-2w_{j}\cdot \;v_{j}^{t}\right)\;\cdot \;\beta_{j}^{t}=\underset{j}{\sum }a_{j}+v_{glc_{-}uptake}\mu_{glc}+v_{biom}^{target}\mu_{biom}-\underset{j}{\sum }e_{j}v_{j}^{min}+\underset{j}{\sum }f_{j}v_{j}^{max};\\ \;\underset{t}{\sum }\beta_{j}^{t}=1\;\forall j;\;\underset{j}{\sum }\underset{t}{\sum }S_{ij}v_{j}^{t}\beta_{j}^{t}=0\;\;\forall i;\;\underset{t}{\sum }v_{glc}^{t}\beta_{glc}^{t}=v_{glc_{-}uptake};\;\underset{t}{\sum }v_{biom}^{t}\beta_{biom}^{t}\geq v_{biom}^{target};\\ \;\underset{t}{\sum }-v_{j}^{t}\beta_{j}^{t}\geq -v_{j}^{max}\cdot y_{j}\;\forall j;\;\;\underset{t}{\sum }v_{j}^{t}\beta_{j}^{t}\geq v_{j}^{min}\cdot y_{j}\forall j;\\ \;a_{j}+\underset{i}{\sum }S_{ij}v_{j}^{t}b_{i}-v_{j}^{t}c_{j}+v_{j}^{t}d_{j}\leq {v_{j}^{t}}^{2}-2w_{j}v_{j}^{t}\;\;\forall j,t,j\neq glc,biom,chemical;\\ \;v_{glc}^{t}\mu_{glc}+a_{glc}+\underset{i}{\sum }S_{i,glc}v_{glc}^{t}b_{i}-v_{glc}^{t}c_{glc}+v_{glc}^{t}d_{glc}\leq \;{v_{glc}^{t}}^{2}-2w_{glc}v_{glc}^{t}\forall t;\\ \;v_{biom}^{t}\mu_{biom}+a_{biom}+\underset{i}{\sum }S_{i,biom}v_{biom}^{t}b_{i}-v_{biom}^{t}c_{biom}+v_{biom}^{t}d_{biom}\leq {v_{biom}^{t}}^{2}-2w_{biom}v_{biom}^{t}\forall t;\\ \;a_{chemical}+\underset{i}{\sum }S_{i,chemical}v_{chemical}^{t}b_{i}-v_{chemical}^{t}c_{chemical}+v_{chemical}^{t}d_{chemical}\leq 0\;\forall t;\\ \;-My_{j}\leq e_{j}\leq My_{j},\;c_{j}-M\left(1-y_{j}\right)\leq e_{j}\leq c_{j}+M\left(1-y_{j}\right)\;\forall j;\\ \;-My_{j}\leq f_{j}\leq My_{j},\;d_{j}-M\left(1-y_{j}\right)\leq f_{j}\leq d_{j}+M\left(1-y_{j}\right)\;\forall j;\\ \;\mu_{biomass}\geq 0;c_{j}\geq 0,\;d_{j}\geq 0\;\;\forall j;\;\beta_{j}^{t}\geq 0\;\forall j,t. \end{array}$$

This final single-level MILP problem can be solved effectively by professional solvers, such as CPLEX [17]. We note that our new MOMA-based knockout optimization problem has a larger problem size with a larger number of variables and constraints as multiple linear functions are used to approximate the inner quadratic function.

### Adaptive strategy

We have shown that we can effectively solve the linearized bi-level programming problem in the previous section. However, due to the linearization of the original quadratic MOMA objective function, the obtained result for a given linearization scheme is an approximate solution but not exact. In addition, the closeness to the exact optimal solution is directly determined by the number of segments for each flux to approximate the quadratic function $v_{j}^{2}$. In order to obtain the exact optimal solution to the original bi-level programming problem, we adopt an adaptive strategy, in which piecewise linearization is implemented adaptively from the coarse to fine levels. As the original inner problem is to minimize the quadratic MOMA objective function, which is convex. It is easy to prove that the approximate optimal solution for a given linearization will have each flux *v_j _*fall within one segment. In other words, for each flux *v_j_*, piecewise variables $\beta_{j}^{t}$ only have either one (at endpoints) or two adjacent non-zero values for the approximate solution as illustrated in Figure 1.

When we have only one non-zero value within all the piecewise variables $\beta_{j}^{t}$, we obtain the exact optimal solution as the linearized objective function has the exact same value at these segment endpoints. This naturally leads to an adaptive solution strategy to solve the original bi-level programming problem. We start with a coarse linearization with a small number of segments for each flux *v_j _*and solve the single-level MILP problem for this given linearization. We can compute the objective function value difference for the inner problem for the obtained solution as:

(5) $$\Delta_{j}={\left(v_{j}^{t}-w_{j}\right)}^{2}\beta_{j}^{t}+{\left(v_{j}^{t+1}-w_{j}\right)}^{2}\beta_{j}^{t+1}-{\left(v_{j}^{t}\beta_{j}^{t}+v_{j}^{t+1}\beta_{j}^{t+1}-w_{j}\right)}^{2}.$$

Based on the differences and the state of vector ***β_j _***for all flux values, we adaptively add new piecewise linear segments to better approximate the corresponding contributions from each reaction flux to the quadratic objective function in the inner problem. By repeating the above procedure as shown in Figure 1(B-D), we can iteratively solve the problem by adaptively improve the piecewise linearization from coarse to fine levels until adding pieces does not change the objective value. If every Δ*_j _*is less than a very small number ***ϵ ***and every maximum value in ***β_j _***is larger than a constant number *θ *that is close to 1, we can say the algorithm has converged. To speedup the algorithm, the knockouts from previous iteration are used to get a low bound for the MILP problem. Algorithm 1 provides the pseudo code for our adaptive linearization solution strategy to identify optimal knockout strategy for biochemical overproduction under the MOMA constraint.

**Algorithm 1 **Adaptive bi-level MOMAKnock.

Initialize variables.

Initialize the piecewise linearization with k pieces

repeat

   Solve the inner primal problem based on previous knockouts to get a low bound objL;

   Solve the MILP problem with the low bound objL;

   **for **Each flux *j ***do**

      Compute Δ*_j_*.

      **if **Δ*_j _*>*ϵ or *$\underset{t}{\text{max}}\beta_{j}^{t}<\theta$**then**

         Add a segment point at $v_{j}^{t^{*}}\beta_{j}^{t^{*}}+v_{j}^{t^{*}+1}\beta_{j}^{t^{*}+1}$; ($\beta_{j}^{t^{*}}$ and $\beta_{j}^{t^{*}+1}$ are nonzero)

      **end if**

      **end for**

**until **Added segments do not improve the objective function

## Results and discussion

### Succinate production on AntCore metabolism network

First, we implement our new adaptive bi-level programming method--MOMAKnock--to derive optimal knockout strategies for a core *E*. *coli *metabolic network model proposed in [18]. In this network, there are 74 chemicals and 75 reactions. All of the data are obtained from the OptKnock software package [13]. In order to have a fair comparison with OptKnock, we take the same settings as in OptKnock, in which succinate is set as the targeted bio-production, the glucose uptake rate is set at a fixed value 100*mmol*/*gDW *· *hr*, and the minimum biomass is set as 5 *mmol*/*gDW *· *hr*. All of the experiments are based on the aerobic condition for this metabolic model. As the glucose uptake rate is fixed, the biomass and product yields are equal to the corresponding flux rates due to the steady-state stoichiometry constraints. The wide-type flux distribution is computed by maximizing the biomass in the FBA framework as stated in methods section. To evaluate the actual knockout performance based on the derived strategies, we utilize the MOMA objective to compute the flux values for suggested knockout strains as it has been demonstrated that the derived flux distributions under this objective agrees well with the laboratory observations [11]. Both OptKnock and MOMAKnock are tested by setting the knockout number *K *from 2 to 5. Table 1 and 2 summarize the results from OptKnock and MOMAKnock, respectively. The succinate and biomass flux values from each knockout model (OptKnock and MOMAKnock) as well as the corresponding MOMA flux distributions for suggested knockout strains are listed in each table. The *L*_2 _distance from the optimal knockout flux values to wild-type steady-state flux values is denoted by "${∥v-w∥}_{L_{2}}$".

**Table 1:** Results for knockout strains derived by OptKnock on the core E. coli metabolic network

		OptKnock		MOMA Flux		
			
K	Knockouts	Succi	Biomass	Succi	Biomass	${∥v-w∥}_{L_{2}}$
2	kdpg→ pyr + gap (or 6pg→kdpg), fadh2 + 0.5o2→2atp (or suc→ fum + fadh2)	102.98	14.36	26.32	13.18	398.75
3	g6p → 6pg + nadph, 3pg+glu→ser+akg+nadh, nadh → nadph	121.02	7.06	24.45	5.23	633.25
4	g6p → 6pg + nadph, dhap → gap, fadh2 + 0.5o2→2atp (or suc→fum + fadh2), glyc → glyc(ext)	118.71	5.00	84.56	5.00	482.70
5	pep → pyr + atp, mal→ pyr+co2 + nadph, dhap + nadh → glyc3p, glyc3p → glyc, fadh2 + 0.5o2→2atp (or suc→fum + fadh2)	126.33	10.91	38.73	12.75	518.65

**Table 2:** Results for knockout strains derived by MOMAKnock on the core E. coli metabolic network

		MOMAKnock		MOMA Flux		
			
K	Knockouts	Succi	Biomass	Succi	Biomass	${∥v-w∥}_{L_{2}}$
2	6pg→ ru5p+co2+nadph, suc→ fum + fadh2 (or fadh2 + 0.5o2→2atp)	54.41	13.44	40.25	12.65	124.86
3	6pg→ru5p+co2+nadph, fadh2+0.5o2 → 2atp (or suc→ fum + fadh2), ser→ gly + meethf	54.98	12.08	45.71	11.80	157.67
4	pep→ pyr + atp, g6p→ 6pg+nadph, 6pg→ kdpg (or kdpg→ pyr + gap), fadh2 + 0.5o2→2atp (or suc→ fum + fadh2)	57.75	11.24	52.73	10.76	318.52
5	pep → pyr + atp, g6p → 6pg + nadph, 6pg→ kdpg (or kdpg→ pyr + gap), fadh2+0.5o2→2atp (or suc→ fum + fadh2), nadh → nadph	65.25	7.90	53.31	7.65	352.26

Based on the results from OptKnock in Table 1 we can see that the objective function values for the targeted succinate production are indeed high with the biomass maximization assumption as constraints. For example, when the knockout number *K *= 2, OptKnock can achieve as high as over 72.44 percent of the theoretical maximum succinate flux value 142.16 *mmol*/*gDW *· *hr *for its optimal solution. However, when we evaluate the actual flux values under the MOMA objective, the resulting succinate flux value drops to as low as 18.51 percent. Similarly, for *K *= 3 and 5, OptKnock also derives high succinate flux values under the biomass maximization assumption while the actual values drop significantly in suggested knockout strains under the MOMA objective. When *K *= 4, removing four reactions leads to the optimal succinate flux value at 118.71 *mmol*/*gDW *· *hr*. The suggested knockout strategies maintain to obtain a high value as high as 84.56 *mmol*/*gDW *· *hr *for succinate production in the MOMA flux distribution. However, we notice that the corresponding biomass flux values in both OptKnock and MOMA flux distributions are at 5.00 *mmol*/*gDW *· *hr*, which is the minimum biomass flux value set in our experiments to guarantee living cells. Hence, we believe that the derived knockout strain may not be robust, which does not lead to practically feasible knockout strategies but causes the death of cells. We investigate the suggested knockout reactions when *K *= 3 and 4 as the MOMA biomass flux value when *K *= 3 reaches 5.23 *mmol*/*gDW *· *hr*, close to the minimum value. When *K *= 3, the most important Transhydrogenation reaction (nadh → nadph) that produce nadph (Nicotinamide adenine dinucleotide phosphate - reduced) is removed. When *K *= 4, one Glycolysis reaction (dhap → gap) that produces most portion of gap is removed. Both nadph and gap are important precursors in the biomass reaction. Removing these reactions causes the reduction of biomass flux values.

Table 2 summarizes the results from MOMAKnock. We first note that the MOMA flux distributions for all the suggested knockout strategies in fact have the corresponding succinate flux values that are consistently similar to objective function values in MOMAKnock without significant drops. Due to this, although the derived objective function values form OptKnock are higher, the final succinate productions for MOMAKnock suggested knockout strains under the MOMA objective are consistently better than OptKnock suggested knockouts except in the case *K *= 4, in which OptKnock derives an impractical strategy. The optimal succinate flux value from MOMAKnock suggested deletions can improve at least 37.5 percent compared to OptKnock in the MOMA flux distribution. In addition, both the succinate and biomass reaction flux values change smoothly for MOMAKnock strategies. Finally, as expected due to the *L*_2 _distance based phenotypic constraints in the inner level of MOMAKnock, we can see that the optimal knock flux distributions from MOMAKnock is always closer to the wild-type flux distribution compared to OptKnock suggested knockouts.

Biologically, it is interesting to note that our MOMAKnock indeed identifies relevant reactions as suggested knockout reactions. For example, when the knockout number *K *is 2, one of the suggested knockout reactions is to eliminate the reaction that decompose the succinate (suc), and another one is to remove the reactions that involve competing byproduct metabolism for succinate such as 6-Phospho-D-gluconate (6pg) and Ribulose 5-phosphate (ru5p). With *K *= 3, MOMAKnock adds one additional knockout reaction to the previously identified ones based on the *K *= 2 case, which leads to the increase of succinate production to 32.15% of its theocratical maximum value. When *K *= 4, besides the reactions that consume succinate and the competing reactions, the reaction that decompose Phosphoenolpyruvate (pep) is also detected. This increases the succinate to 37.9% of the thearetical maximum. Finally, when K increases to 5, one more reaction is knocked out, which lead to 53.31 *mmol*/*gDW *· *hr *succinate produce rate. While as mentioned above, this reaction can convert biomass product to biomass precursor, so the deletion causes the reduction of the biomass flux rate.

Based on these preliminary results on this core network model, even though the OptKnock takes the maximizing biomass production as the inner cellular objective, the derived knockout strategies do not always achieve high biomass production when we simulate these knockout strategies under the MOMA objective. Sometimes, these knockout strategies cannot even guarantee the minimum biomass requirement. The reason for this is that the inner optimization in the bi-level framework of OptKnock serves as the additional constraint for the outer optimization problem. The derived optimization procedure first considers the outer problem as the primary objective and then the inner problem is optimized. The simulated low targeted chemical production rates for OptKnock suggested knockouts in the MOMA flux distribution and the abrupt biomass level changes in OptKnock illustrate that the biomass maximization assumption to approximate cellular objectives may not provide robust and reliable metabolic reaction deletion strategies. On the other hand, MOMAKnock approximates the inner cellular objective by the MOMA assumption which assumes that knockout strains stay closer to the corresponding wild-type strains. If this is guaranteed, knockout strains also can achieve appropriate biomass flux values. In fact, as shown in Tables 1 and 2, MOMAKnock not only achieves higher targeted succinate flux values under the MOMA objective but also obtains appropriate biomass flux values within the normal range compared to OptKnock. We also notice that with the increasing *K*, both the targeted succinate flux values and biomass values change smoothly contrasting to the abrupt changes in OptKnock, which may also serve as an evidence that MOMAKnock can help derive more robust knockout strategies with predictable performance.

By comparison with OptKnock on this core *E. coli *metabolic network, it is clear that our MOMAKnock may suggest more practical and robust knockout strategies for optimal bio-productions under phenotypic constraints.

### Succinate production on iAKF1260 network

**Figure 2:**
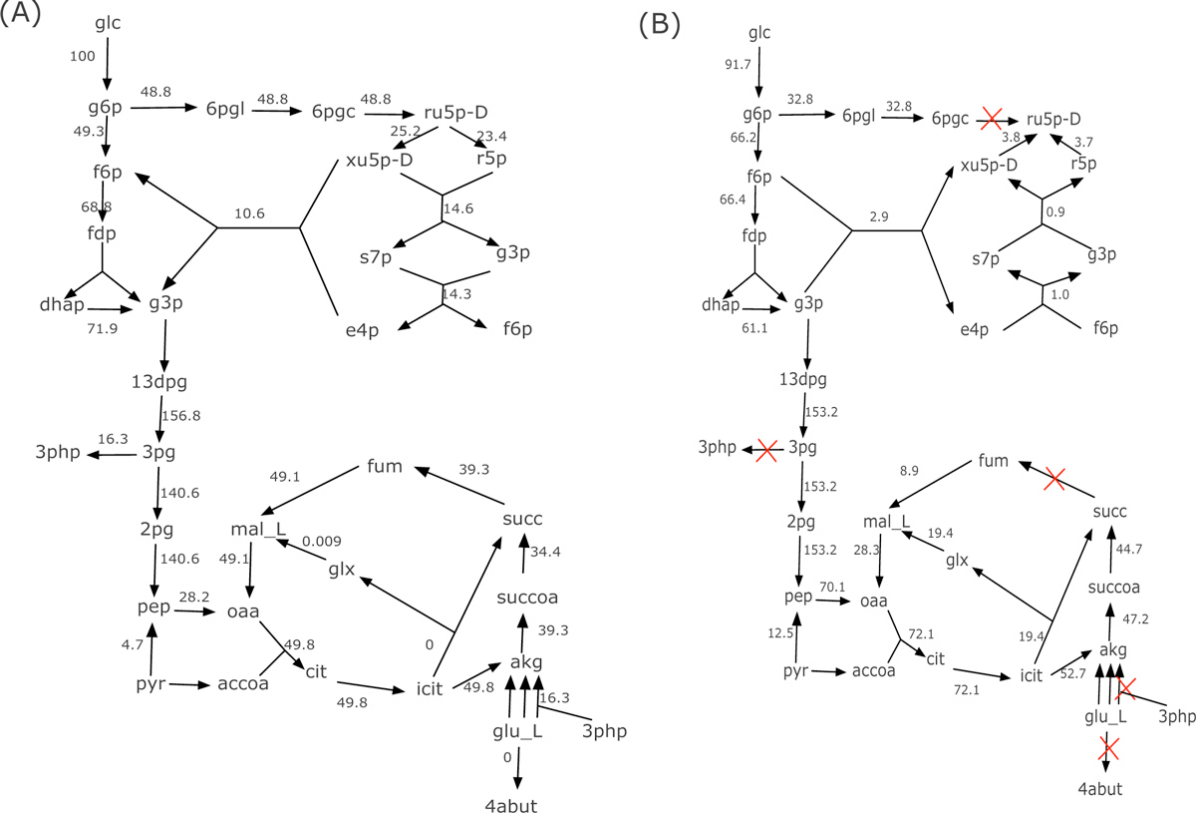
**The MOMA flux distribution: (A) wild-type E. coli network, (B) *K *= 5 MOMAKnock mutant**. (Only a part of the network is presented.)

We further test MOMAKnock on a large *E. coli *metabolic network model--iAF1260 [19], which has 1,658 metabolicals and 2,936 reactions including the pseudo reactions required for the computation model. As in the core network model, succinate is set as the target chemical, the glucose uptake rate is fixed at 100 *mmol*/*gDW *· *hr*, and the minimum biomass is also set to 5 *mmol*/*gDW *· *hr*. All of our experiments are still based on the aerobic environment and all of the data are also from the OptKnock software package [13]. Table 3 provides the results from MOMAknockout for *K *= 3, 4, 5. Figure 2 shows the MOMA flux distribution for the wild-type strain as well as the MOMA flux distribution and the corresponding knockout reactions for the derived knockout strain with *K *= 5.

**Table 3:** Results for knockout strains derived by MOMAKnock on the iAF1260 E. coli metabolic network

		MOMAKnock		MOMA Flux		
			
K	Knockouts	Succi	Biomass	Succi	Biomass	${∥v-w∥}_{L_{2}}$
3	q8+succ→fum+q8h2, 6pgl+h2o→6pgc+h, (2)h2o + o2 + urate → alltn + co2 + h2o2	39.30	5.02	27.45	5.02	906.49
4	q8+succ→fum+q8h2, ac + atp → actp + adp, h2o+methf→10fthf+h, r5p+xu5p-D→g3p+s7p	67.08	5.02	63.23	5.02	402.33
5	q8+succ→fum+q8h2, glu-L+h→4abut+co2, 3pg+nad→3php+h+nadh, 3php+glu-L→akg+pser-L, 6pgc+nadp→co2+nadph+ ru5p-D	74.94	5.02	66.67	5.02	464.76

From Figure 2 and Table 3 we can see that, similar as in the core network model, MOMAKnock suggests knockout reactions in this large network that mostly contain the reactions that directly consume succinate, which include the succinate dehydrogenase reaction (SUCDi), as well as the competing reactions that may consume the precursors for succinate production, such as 6-phosphogluconolactonase (PGL), transketolase (TKT1) and phosphogluconate dehydrogenase (GND). The knockouts also contain some nonintuitive reactions as the final network dynamics is determined globally due to highly complex interactions among different reactions. When *K *= 5, the succinate production can achieve as high as 79.73% of the theocratical maximum rate (83.62*mmol*/*gDW *· *hr*), which demonstrates that our MOMAKnock can serve as a computational tool for deriving potentially effective and robust knockout strategies.

We notice that in Table 3 all of the biomass value is near 5 *mmol*/*gDW *· *hr*, which is the minimum biomass value set in the all of the tests. However, in this large network, the theoretical maximum biomass is 9.657*mmol*/*gDW *· *hr*. Experiments shows that if the the succinate dehydrogenase reaction (SUCDi) is recovered from knockouts, we can get higher biomass but the succinate can drop to as low as 10 *mmol*/*gDW *· *hr*. As shown in Figure 2, the reason for this is that the SUCDi reaction is the only direct pathway that can convert succinate back to some biomass precursors. Due to this reason, MOMAKnock derives the suggested knockout strategies, which try to find a point that can balance the succinate and biomass production.

## Conclusions

In this paper, we have proposed a new bi-level programming optimization framework to identify optimal knockout strategies for maximum targeted bio-productions under the phenotypic constraints approximated by the MOMA assumption. A novel adaptive piecewise linearization solution strategy has been developed to efficiently solve this new mixed integer quadratic bi-level programming problem. The preliminary experiments on both the core *E. coli *metabolic network model [18] and the large-scale iAF1260 *E. coli *metabolic network model [19] have demonstrated its potential in *in silico *metabolic engineering to help derive effective genetic or metabolic intervention strategies through genome-scale network dynamic analysis based on the FBA framework. To better approximate the phenotypic constraints for knockout strains, we have take the MOMA assumption instead of the maximal growth assumption as in OptKnock to model the underlying cellular objective. Based on the obtained results on two network models, it is clear that MOMOKnock derives improved knockout strategies under the MOMA objective, which are more robust and practical.

Our new bi-level MOMAKnock model can serve as an alternative method with slightly higher computational complexity to OptKnock for *in silico *metabolic engineering. In addition to that, according to different cellular objective assumptions, we can formulate different inner problems as phenotypic constraints in this bi-level programming framework to derive optimal intervention strategies under different conditions. Our future research will focus on developing and testing such new models for large-scale metabolic networks. For example, as ROOM [12] suggests, constraining on the number of significantly modified flux values can lead to better predictions for knockout strains through long-term evolutionary pressure. The corresponding mathematical formulation can be done by replacing the *L*_2 _distance objective function in MOMA by either *L*_0 _or *L*_1 _norm, which will lead to different bi-level optimization problems. We will develop corresponding solution strategies to solve this category of bi-level problems for large-scale networks and compare their performances with respect to the efficacy and robustness of the correspondingly derived intervention strategies.

## Competing interests

The authors declare that they have no competing interests.

## Authors' contributions

Conceived and designed the experiments: XQ. Designed and Implemented the algorithm: SR, BZ, XQ. Performed the experiments: SR. Analyzed the results: SR, BZ, XQ. Wrote the paper: SR, BZ, XQ.

## Declarations

The publication costs for this article were funded by the corresponding author's institution.

This article has been published as part of *BMC Bioinformatics *Volume 14 Supplement 2, 2013: Selected articles from the Eleventh Asia Pacific Bioinformatics Conference (APBC 2013): Bioinformatics. The full contents of the supplement are available online at http://www.biomedcentral.com/bmcbioinformatics/supplements/14/S2.
